# Targeting PBK/TOPK decreases growth and survival of glioma initiating cells in vitro and attenuates tumor growth in vivo

**DOI:** 10.1186/s12943-015-0398-x

**Published:** 2015-06-17

**Authors:** Mrinal Joel, Awais A. Mughal, Zanina Grieg, Wayne Murrell, Sheryl Palmero, Birthe Mikkelsen, Hege B. Fjerdingstad, Cecilie J. Sandberg, Jinan Behnan, Joel C. Glover, Iver A. Langmoen, Biljana Stangeland

**Affiliations:** Vilhelm Magnus Laboratory for Neurosurgical Research, Department of Neurosurgery and Institute of Surgical Research, Oslo University Hospital, Oslo, Norway; Laboratory of Neural Development and Optical Recording (NDEVOR), Department of Physiology, Institute of Basic Medical Sciences, University of Oslo, Oslo, Norway; Oslo University Hospital, SFI-CAST Biomedical Innovation Center, Oslo, Norway; Norwegian Center for Stem Cell Research, Department of Immunology and Transfusion Medicine, Oslo University Hospital, Oslo, Norway; Present Address: Department of Radiation Biology, Institute for Cancer Research, Norwegian Radium Hospital, Oslo University Hospital, Oslo, Norway

**Keywords:** PBK/TOPK, Glioblastoma, Tumor, shRNA, Inhibitor, Cell culture

## Abstract

**Background:**

Glioblastomas are invasive therapy resistant brain tumors with extremely poor prognosis. The Glioma initiating cell (GIC) population contributes to therapeutic resistance and tumor recurrence. Targeting GIC-associated gene candidates could significantly impact GBM tumorigenicity. Here, we investigate a protein kinase, PBK/TOPK as a candidate for regulating growth, survival and *in vivo* tumorigenicity of GICs.

**Methods:**

PBK is highly upregulated in GICs and GBM tissues as shown by RNA and protein analyses. We knocked down *PBK* using shRNA vectors and inhibited the function of PBK protein with a pharmacological PBK inhibitor, HITOPK-032. We assessed viability, tumorsphere formation and apoptosis in three patient derived GIC cultures.

**Results:**

Gene knockdown of *PBK* led to decreased viability and sphere formation and in one culture an increase in apoptosis. Treatment of cells with inhibitor HITOPK-032 (5 μM and 10 μM) almost completely abolished growth and elicited a large increase in apoptosis in all three cultures. HI-TOPK-032 treatment (5 mg/kg and 10 mg/kg bodyweight) *in vivo* resulted in diminished growth of experimentally induced subcutaneous GBM tumors in mice. We also carried out multi-culture assays of cell survival to investigate the relative effects on GICs compared with the normal neural stem cells (NSCs) and their differentiated counterparts. Normal NSCs seemed to withstand treatment slightly better than the GICs.

**Conclusion:**

Our study of identification and functional validation of PBK suggests that this candidate can be a promising molecular target for GBM treatment.

**Electronic supplementary material:**

The online version of this article (doi:10.1186/s12943-015-0398-x) contains supplementary material, which is available to authorized users.

## Background

Glioblastoma multiforme (GBM) is both the most common and the most malignant primary brain tumor. Despite aggressive treatment with surgical resection, chemotherapy and radiotherapy, median survival is only one year [1, 2].

Several studies suggest that GBM is hierarchically organized. The apex of this hierarchy consists of a subgroup of cells functionally defined by the ability to self-renew and to reproduce the tumor of origin in orthotopic transplantation models [3–5]. Although it remains unclear whether a cancer cell with stem-like properties initiates GBM, this subgroup of GBM cells shares a number of properties with neural stem/ progenitor cells from the normal adult human brain, and are most commonly referred to as glioma initiating cells (GICs). GICs are relatively unaffected by irradiation and chemotherapy and are thought to be important in therapeutic resistance and tumor recurrence [6–8]. Identification of therapeutic targets in GICs thus offers promise for the development of novel treatments for GBM [9–11].

To this end, we have performed comparative gene expression profiling in GICs and in stem cells from the normal adult human brain and identified a number of genes and pathways that are differentially regulated in GICs [12] (Stangeland *et al.,* submitted). The PDZ-binding kinase/T-LAK cell-originated protein kinase (*PBK/TOPK*) was highly up-regulated in GICs from all patients we studied, also confirmed at the protein level (Stangeland *et al.,* submitted). Protein kinases play key roles in the regulation of intracellular pathways that control cell growth and survival [13] and are often involved in the precipitation of malignancy. Inhibition of protein kinases is therefore considered a potentially fruitful approach for arresting the growth of tumors [14–16]. Previously, PBK/TOPK, a serine-threonine kinase and a member of MAPKK family, has been shown to play important roles in both normal and cancer cells [17–22]. Among normal cell types, PBK/TOPK is expressed in highly proliferating cells such as spermatocytes, in several fetal tissues as well as in neural stem and progenitor cells [18, 23]. Studies of neural progenitor cells show that phospho-PBK/TOPK is detected specifically in M-phase in association with condensed chromatin [18]. PBK/TOPK acts as a MAP kinase kinase by phosphorylation of P38 mitogen-activated protein kinase (MAPK) [17, 24] and is active during the mitotic phase of the cell cycle [17]. During mitosis, PBK/TOPK and cdk1/cyclin B1 complex promote cytokinesis through phosphorylation of a protein regulator of cytokinesis 1 (PRC1) [25–27] and a positive feedback loop between PBK/TOPK and ERK2 promotes uncontrolled proliferation [21]. There are also studies suggesting a role for PBK/TOPK in the sensing and repair of DNA damage through phosphorylation of histone H2AX [17, 22, 27]. Together these studies suggest that PBK/TOPK may play an important role in linking extracellular signals to signaling pathways that influence cell proliferation.

The goal of the present study was to investigate the functional significance of PBK/TOPK up-regulation in GBM. We show that knockdown of *PBK/TOPK* expression using lentiviral short hairpin RNA (shRNA) vectors, as well as inhibition by a specific antagonist HI-TOPK-032 [28], reduces cell viability and sphere formation *in vitro*. HI-TOPK-032 treatment also leads to a massive increase in apoptotic cells. Further, treatment with HI-TOPK-032 *in vivo* results in a significant dose-dependent decrease of tumor growth. We also investigated the relative effects on tumor cells compared with normal brain stem cells and their differentiated counterparts. Normal NSCs seemed to withstand treatment slightly better than GICs and both normal- and tumor-derived differentiated cells fared better than GICs. PBK should therefore be investigated further as a putative target for molecular therapy in GBM.

## Results

### PBK is upregulated in seven different patient-derived GIC cultures

**Fig. 1:**
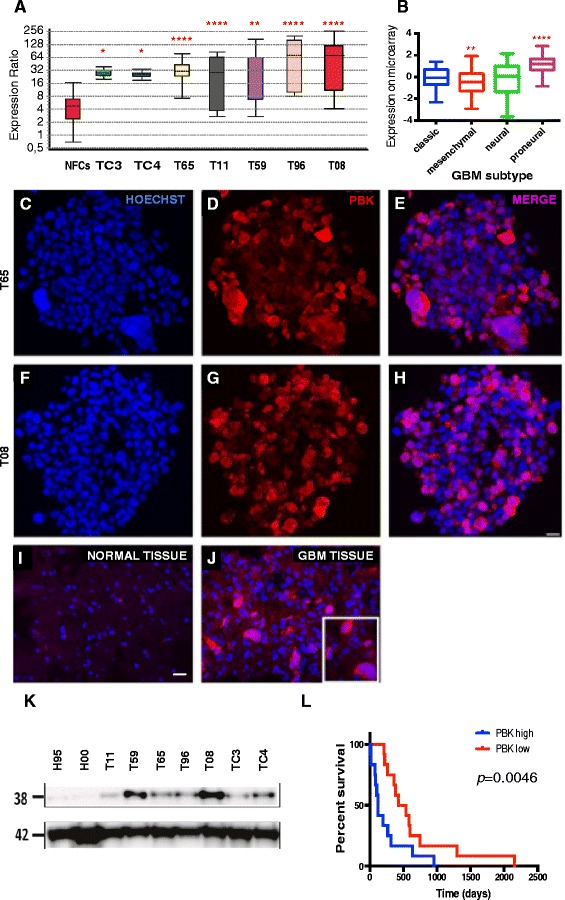
Expression of PBK in different GIC cultures. **a** Expression of *PBK* gene in NFCs and seven different GIC cultures. Box plot shows significantly increased expression levels of *PBK* in GIC cultures. Relative expression of *PBK* was calculated using normal NSCs from the adult human brain as a reference (Relative expression of *PBK* in NSCs = 1, not shown). Relative expression of *PBK* was not significantly increased in NFCs (*p* = 0.057). **b** Expression of *PBK* gene in GBM tissue samples from TCGA. *PBK* was significantly up-regulated in proneural and down-regulated in mesenchymal subtypes of GBM. *PBK* expression in different subtypes was performed using the classical subtype as a reference. Common for A and B: The bottom and top of each box in box and whisker plots indicate the 25th and 75th percentile (the lower and upper quartiles, respectively), and the band near the middle of the box represents the 50th percentile (the median) (**c**-**h**) Immunostaining of cryosections of GIC spheres (T65 and T08) shows expression of PBK (red) and its cellular co-localization with Hoechst (blue). Scale bar = 20 μm. **i**, **j** Immunolabeling of a GBM tissue with PBK antibody shows its extensive presence in the GBM tissue compared to the normal cortex brain tissue. Scale bar = 20 μm. **k** Western blot analysis done on seven GIC cultures shows a clear upregulation of PBK as compared to that in the two normal NSC cultures. ACTB was used as a control. **l** Kaplan-Meier graph showing patient survival in mesenchymal subtype of GBM (TCGA). The survival times of GBM patients with highest (30 %) and lowest (30 %) expression of *PBK* were compared (*p* = 0.0046 according to Gehan-Breslow-Wilcoxon test). Increased expression of *PBK* correlated negatively with patient survival. Asterisks correspond to p values and indicate level of significance: * = (p ≈ 0.01-0.05), ** = (p ≈ 0.001-0.01) and **** = (p < 0.0001)

To assess PBK expression in GBM, we first investigated the mRNA and protein levels of PBK in GIC cultures derived from human brain tumor and in normal samples. We first compared mRNA levels in seven GIC cultures and in the neural fetal progenitor cell line (NFCs, official name: ReNcell, Millipore) to those in two NSC cultures, using qPCR. qPCR analysis showed that *PBK* mRNA expression in GIC cultures is much higher than in NSCs (Fig. 1a, Additional file 1: Table S1). We have also assessed the expression of *PBK* in GBM tissue samples from TCGA. This analysis showed that PBK was significantly up-regulated in the proneural and down-regulated in the mesenchymal subtypes of GBM (Fig. 1b).

Immunostaining of cryosections of GBM tumorspheres (T65 and T08) confirmed high protein expression and demonstrated an extensive cytoplasmic location of PBK in tumorspheres derived from these GIC cultures (Fig. 1c-h). Immunostaining in the respective primary GBM tissues revealed a higher level of PBK than in surrounding normal cortical tissue, indicating that the high level of expression is not a culturing artifact (Fig. 1i, j). Western blot analysis confirmed the elevated expression of PBK in different GIC cultures compared to two different normal NSC cultures (Fig. 1k). The survival times of GBM patients (TCGA) with the highest and lowest expression of *PBK* were compared using Kaplan-Meier estimator. This analysis showed that the increased expression of PBK correlated negatively with the survival of mesenchymal subtype patients (Fig. 1l). The survival results for other subtypes were not significant.

Taken together, these results indicate that PBK is strongly upregulated both in primary GIC cultures and in GBM tissue samples. These observations are consistent with several studies that have demonstrated an increased expression of PBK in various malignancies [17, 19, 21, 24, 29–32]. These results prompted us to further explore the role of PBK in GBM growth and survival using two approaches to inhibit its function: knockdown by shRNA and pharmacological blockade of interaction with targets.

### Knockdown of *PBK* with shRNA reduces viability and sphere formation, and can increase apoptosis

**Fig. 2:**
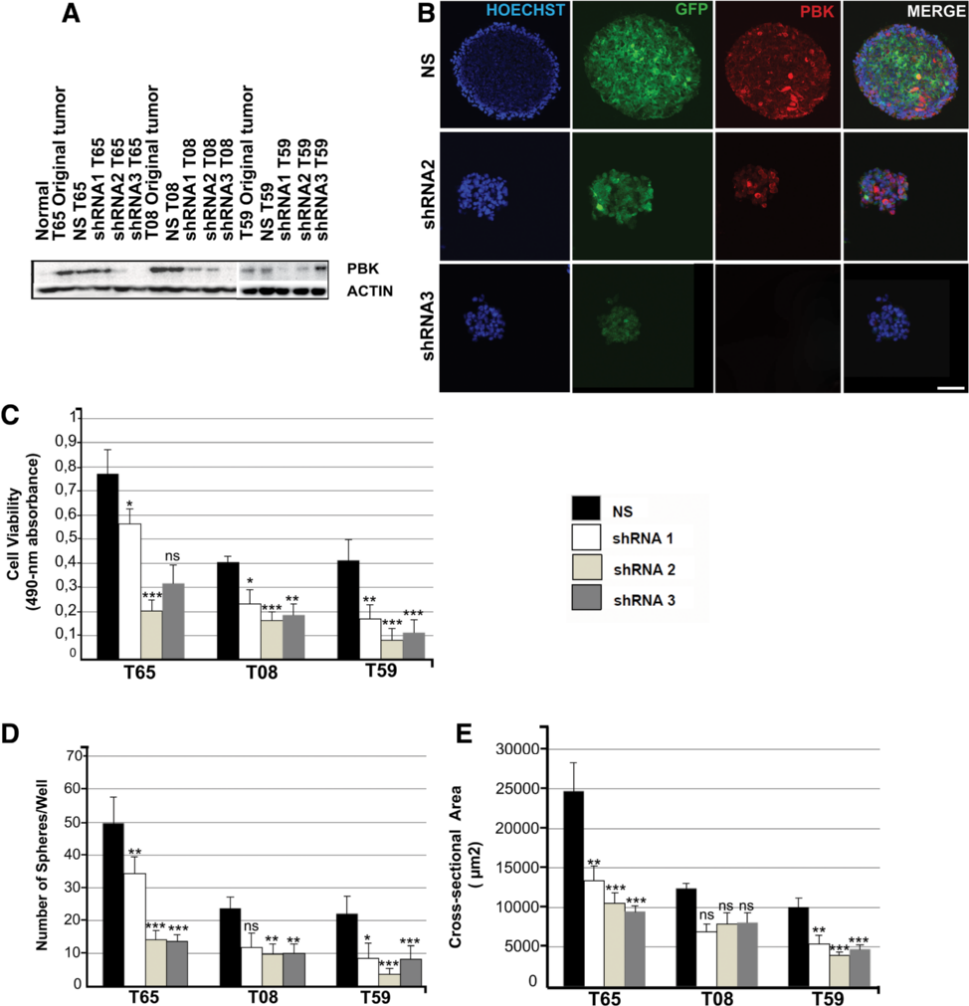
*PBK* knockdown with lentiviral shRNAs led to reduction in viability and sphere forming capacity in GICs. **a** Western blot analyses demonstrated down-regulation of PBK protein level in T65, T08 and T59 shRNAs 1, 2 and 3 cell lines compared to non-silencing cells and the original tumor. ACTB was used as a control. **b** Immunofluorescence analysis of cryosections of GIC spheres transduced with shRNA 2 and 3 compared to the control, shows the loss of PBK protein in the cells present in the spheres. This loss of PBK directly correlated to the loss of GFP. Scale bar = 20 μm. **c** Quantitative analyses of *PBK* knockdown cell lines of different GICs showed a significant decrease (*n* = 5 in each assessment) in viability of knockdown cells compared to the non-silencing controls. **d**, **e** Analysis of sphere formation capacity - Sphere numbers and sphere areas of the *PBK* knockdown cell lines were also diminished as compared to the non-silencing controls. *PBK* knockdown led to an efficient decrease in the sphere formation capacity of GICs. Error bars = SD; *n* = 5. Asterisks correspond to p values and indicate level of significance: * = (p ≈ 0.01-0.05), ** = (p ≈ 0.001-0.01) and **** = (p < 0.0001)

We created a knockdown of *PBK* in GICs by using RNAi technology (Thermo Scientific, Open Biosystems). We used three different shRNA constructs directed against *PBK* mRNA and a non-silencing shRNA as a control. These shRNA constructs were used to knock down *PBK* in GIC cultures (T59, T65 and T08). The knockdown efficiency was monitored by qPCR (Additional file 2: Figure S1 and Additional file 3: Table S2) and Western blot analysis, which showed that shRNAs led to efficient protein knockdowns in several cultures (Fig. 2a). The strongest PBK protein knockdowns were obtained with shRNA1 in T59 and shRNA3 in T65 and T08 (Fig. 2a). In GIC culture T59 treated with shRNA3 we could not detect *PBK* knockdown at mRNA level (Additional file 2: Figure S1). However, we detected slightly increased levels of PBK protein (Fig. 2a). To investigate the degree of linear dependence between the knockdown efficiencies at the transcript and protein levels we compared the qPCR data to the quantified western data using Pearson product–moment correlation coefficient (PPMCC *r*) calculation. Correlation was good (*r* = 0.68), very good (*r* = 0.77) and excellent (*r* = 0.97) for T59, T08 and T65 respectively.

Immunostaining of cryosections from T08 tumorspheres treated with shRNA2 and shRNA3 further confirmed the decrease of PBK expression compared to non-silencing shRNA controls. PBK immunostaining was strongly reduced by shRNA2 and completely abolished by shRNA3 (Fig. 2b left to right), essentially mirroring the results obtained with Western blotting.

The above results indicate that an efficient knockdown of PBK at the protein level can be obtained using shRNA vectors. Therefore, to elucidate the role of PBK in cellular functions involved in the growth and survival of GIC cultures, we performed viability, sphere formation and apoptosis assays on the three GIC cultures (T59, T65 and T08) featuring PBK knock-down.

Viability, number of spheres formed, and sphere size were all reduced, in most cases at statistically significant levels, in GIC cultures with all three shRNA constructs as compared to non-silencing shRNA controls (Fig. 2c-e). To calculate the degree of linear dependence between the knockdown efficiencies and the results of the functional assays we used PPMCC. Correlation between the results of the cell viability assay and knockdown efficiency was very good (*r* = 0.82) for T59 and excellent for T65 and T08 (*r* = 0.91 and *r* = 0.93 respectively). Correlation between the number of spheres and the knockdown efficiency was excellent for all three cultures (*r* = 0.9, *r* = 0.93 and *r* = 0.97 in T59, T65 and T08 respectively). Also the size of the spheres correlated nicely to the knockdown efficiency values and ranged from *r* = 0.76 in T65 to *r* = 0.84 in T59 and *r* = 0.99 in T08. Because the decrease in viability was paralleled by decreases in sphere formation and size, it is likely to be due in large part to inhibition of cell proliferation and growth. Since PBK seems to play an important role in the mitotic phase of the cell cycle of some cancer cell types, we also investigated the effect of *PBK* knockdown on cells arrested in the G2/M phase of the cell cycle [30]. Only one of the three GIC cultures was arrested with the three different knockdown constructs in the G2/M phase of the cell cycle (data not shown).

To assess the effect of *PBK* knockdown specifically on cell survival, we quantitated apoptosis by measuring caspase-3 activity. Caspase-3 activity was increased by about 4-fold in T08 by all three shRNAs relative to ns-shRNA, whereas it was unaffected or even reduced in T59 and T65 (Additional file 4: Figure S2). Thus, *PBK* knockdown has stronger effects on proliferation and growth than on cell death in GIC cultures.

### Effects of HI-TOPK-032 on viability, sphere formation, and apoptosis

**Fig. 3:**
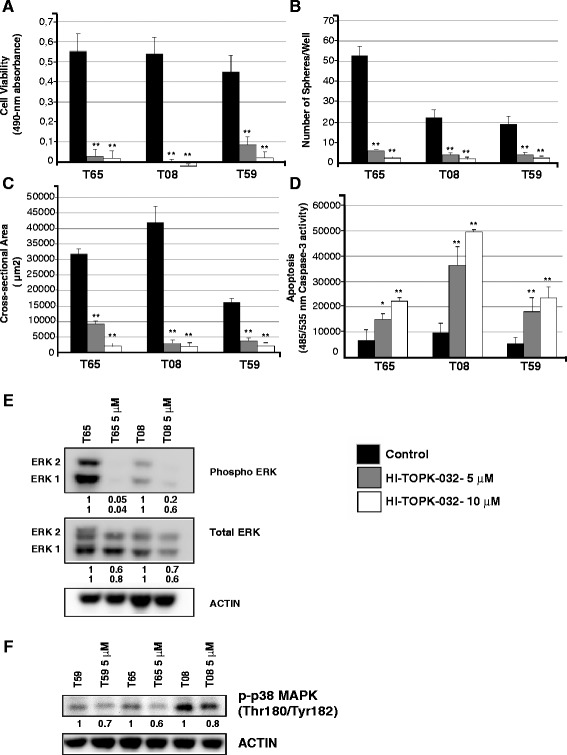
Pharmacological treatment of GICs with HI-TOPK-032 depleted PBK, and reduced their viability and sphere forming capacity. **a** HI-TOPK-032 treated cells exhibited significant decrease in viability. **b**, **c** Sphere formation capacity - Sphere number and sphere area of the PBK treated cells were also evaluated as compared to the untreated cells. PBK inhibition led to an efficient decrease in the sphere formation capacity of GICs. **d** Induction of cell death in HI-TOPK-032 treated cells as compared to untreated ones was significant in all cases (**e**) Western blot showing Total and Phospho ERK in two GIC cultures treated with HI-TOPK-032 versus untreated cultures. **f** Western blot showing phosphorylation status of the p38 MAP kinase (Thr180/Tyr182) in three GIC cultures treated with HI-TOPK-032 versus untreated cultures. The relative expression values were calculated by normalization to ACTB expression and using untreated cultures as references. Error bars = SD; *n* = 5. Asterisks correspond to p values and indicate level of significance: * = (p ≈ 0.01-0.05), ** = (p ≈ 0.001-0.01) and **** = (p < 0.0001)

To examine the effects of HI-TOPK-032 (an agonist that binds to the active site of PBK [28], cells from each of the three GIC cultures were treated with 5 μM and 10 μM HI-TOPK-032, concentrations shown previously to efficiently inhibit PBK in other cancer cell lines [28]. Cells were treated for three days and viability was assessed for a period of 24 h. Viability was powerfully inhibited (more than 90 %) in a dose-dependent manner (Fig. 3a). Sphere formation was assessed in the same cultures after 10 days of culture. Both sphere number and sphere area were also massively reduced (by 80-90 % in all cases; Fig. 3b, c). This was paralleled by a substantial, dose-dependent increase in apoptosis (Fig. 3d). Thus, the massive decrease in viability is clearly due in large part to decreases in proliferation and in cell survival.

We also tested the effect of HI-TOPK-032 (5 μM) on one of the knockdown cell lines (shRNA3) and its non-silencing control for each of the cultures T65, T08, and T59, and found that cells featuring *PBK* knockdown were more resistant than non-silencing control cells to HI-TOPK-032 treatment with respect to cell viability, sphere forming capacity and apoptosis (Additional file 5: Figure S3). These findings indicate that the effect of HI-TOPK-032 is stronger and has a more specific effect in cells containing more PBK protein.

We have also performed an *in vitro* dilution assay using single cell sorting of T08 control and HI-TOPK-032 treated (5 μM) cells. The effect of HI-TOPK-032 on the clonally derived spheres was similar to that seen in the experiments described above (Additional file 6: Figure S7).

### Microarray analysis

Differentially regulated genes were identified using the Rank product analysis and 1 % false discovery rate (FDR). For the analysis of PBK knockdowns; we included shRNA2 and shRNA3 of T08 tumor culture with their corresponding non-silencing controls (all in triplicate). We identified 1585 differentially regulated genes, 963 and 622 being up- and down-regulated, respectively. For the transcriptome analysis of PBK inhibitor–treated samples; T65, T08 and T59 tumor cultures were treated with PBK inhibitor at a concentration of 5 μm (all in triplicate). We identified 2712 differentially expressed genes, 1380 up- and 1332 down-regulated genes compared to the control. Directly comparing the gene lists; we found that 549 genes were overlapping between the PBK-knockdown and the inhibitor-treated groups. These genes were mostly involved in developmental processes and adhesion (Gene Ontology analysis; Additional file 7: Table S3). Pathway analysis was conducted individually for both groups, and interestingly we identified a similar profile of pathways being significantly regulated in both knockdowns and inhibitor-treated cells: metabolic pathways, cell cycle, focal adhesion, pathways in cancer, cell adhesion molecules and ECM-receptor interaction.

In addition to this, we have also analyzed a few cancer-related pathways such as Wnt, Notch and EGF in individual KD and inhibitor treated cultures. We found that several important genes in these pathways were differentially regulated in different cultures compared to their respective controls. Principal component analysis showed a significant differential distribution of Wnt, Notch, and EGF-related genes in shRNA/PBK inhibitor treated cell lines compared to controls (data not shown).

### ERK and p38 MAPK signaling

We next investigated the effect of HI-TOPK-032 on the ERK signaling pathway, which is a direct downstream target of PBK. Cells from two of the GIC cultures were treated with HI-TOPK-032 for three days and then cell lysates were examined by Western blot analysis. Inhibition of PBK by HI-TOPK-032 led to moderate decrement in total ERK1/2 levels and significant reduction in phosphorylated ERK1/2 (Fig. 3e).

PBK is necessary for appropriate activation and function of the p38 MAP Kinase pathway by growth factors [18, 23]. The regulation of p38 phosphorylation status by PBK is mediated via several factors including ERK1/2 [17, 21]. Western blot showed down-regulation of phospho-p38 MAP Kinase (Thr180/Tyr182) in cultures treated with HI-TOPK-032 (Fig. 3f). We also investigated the influence of PBK knockdown on levels of phospho-p38 MAP Kinase. In GIC cultures featuring PBK knockdown the levels of phospho-p38 MAP Kinase (Thr180/Tyr182) were also decreased (Additional file 8: Figure S8).

Taken together, these results suggest that PBK plays a vital role in the growth and survival of GIC cultures and that HI-TOPK-032 is an efficient antagonist of PBK.

### Therapeutic inhibition of PBK attenuates growth of subcutaneous tumor xenografts in vivo

**Fig. 4:**
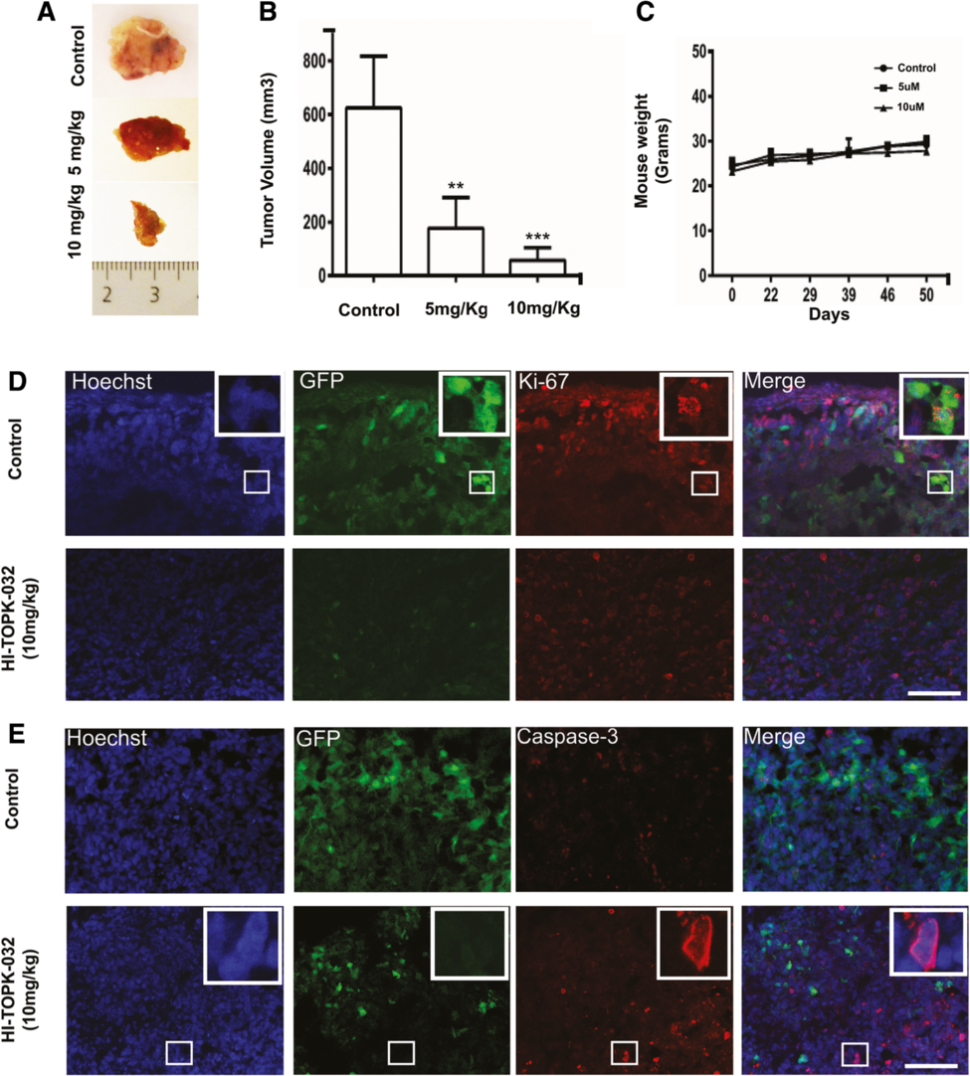
Anti-tumor effects in tumor xenografts upon inhibition of PBK through administration of HI-TOPK-032. **a** T08 tumor cells were subcutaneously transplanted in the right flank of SCID mice. After establishment of tumors, mice were treated three times weekly by intra-tumoral injection of (i) control and (ii) HI-TOPK-032 (5 and 10 mg/kg) suspensions. The tumors were extracted after 4 weeks of treatment and a representative tumor from each group is shown. **b** Mean tumor volume was calculated. Error bars = SD. Mean tumor volumes of HI-TOPK-032 treated tumors (5 and 10 mg/kg) were significantly different than the control group (p < 0.05 and p < 0.005 respectively). **c** HI-TOPK-032 had no effect on mouse body weight. Mice in both treated and untreated groups were weighed once a week. Error bars = SD (**d**, **e**). Immunofluorescence analysis of HI-TOPK-032 (10 mg/kg) treated and control tumor cryosections stained for Ki-67 and cleaved caspase-3 expression. Scale bar = 20 μm

To further investigate the effectiveness of PBK inhibition we performed an experimental therapy approach using exogenously delivered PBK inhibitor to severe combined immune-deficient (SCID) mice with subcutaneous tumors. To establish these tumors we injected T08 cells subcutaneously into the flank, and then injected the inhibitor locally into the resultant tumor starting three weeks later. Mice were injected with vehicle control or HI-TOPK-032 at 5 or 10 mg/ kg three times a week over a period of 28 days. This treatment regimen significantly inhibited tumor growth (mean tumor volume) by more than 3–5 fold relative to controls in a dose-dependent manner (Fig. 4a, b). Mice appeared to tolerate the intra-tumor injection of HI-TOPK-032 without any signs of toxicity or loss of body weight (Fig. 4c). Thus, HI-TOPK-032 appears to arrest tumor growth through inhibition of PBK *in vivo*.

Immunohistochemical analysis of the proliferation marker Ki67 (Fig. 4d) and the apoptosis marker cleaved caspase-3 (Fig. 4e) showed a reduction in the proportion of Ki67-positive cells and a slight increase in apoptosis in HI-TOPK-032-treated tumors compared with controls. These findings support the interpretation that the observed decline of tumor growth in the inhibitor-treated tumors *in vivo* is caused by a reduction of proliferation and an increase in apoptosis.

### Effects on wide range of GBM patient samples: *in vitro* simulation of treatment with inhibitor

**Fig. 5:**
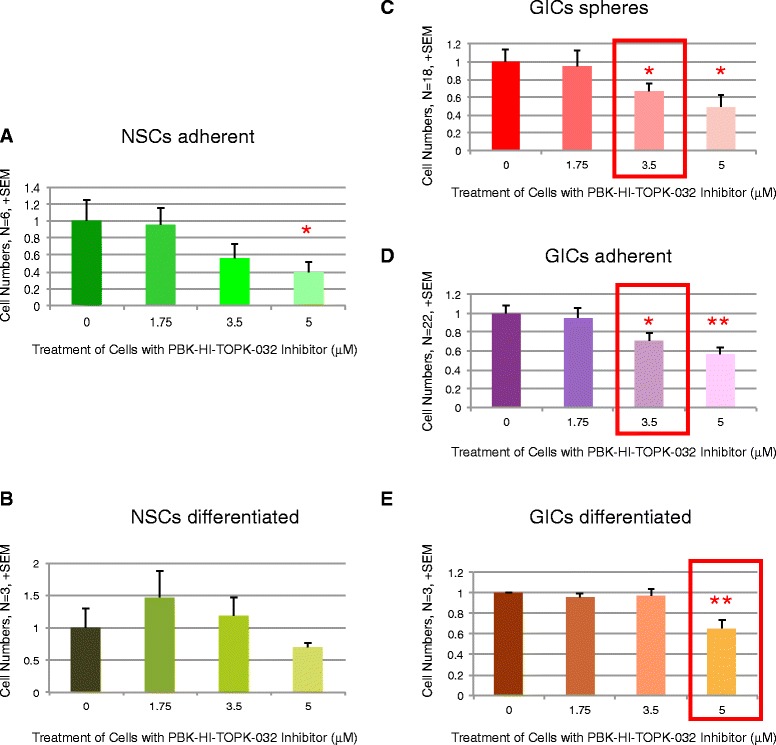
Effects on patient cells: *In vitro* simulation of treatment with inhibitor. Net live cells expressed as fold of cell numbers for cultures with vehicle only. HI-TOPK-032 was included in cultures at various concentrations. There was a trend of dose dependent reduction with concentration in all cultures (2-Way Anova: *p* = 0.0031; A, *n* = 22; B, *n* = 18; C, *n* = 6; D, *n* = 3; E, *n* = 3. Bar: + SEM). At an inhibitor concentration of 3.5 μM decreases in cell numbers were significant in GIC cultures (**c**-**d**) but not in NSC cultures (**a**) thus presenting a narrow therapeutic window (indicated with red rectangles). Using individual T-tests to compare dose concentrations, normal NSCs (**a**), normal differentiated cells (**b**) and tumor differentiated cells (**e**) were not significantly reduced at 3.5 μM when compared to 0 μM). At an inhibitor concentration of 3.5 μM both groups of tumor stem cells experienced a significant reduction in net live cells. However, at an inhibitor concentration of 5 μM decreases in cell numbers were significant in differentiated GIC cultures (**e**) but not in differentiated NSC cultures (**b**) thus presenting a narrow therapeutic window (indicated with red rectangle)

As a clear effect of PBK blockade had now been established by various means and as the *in vivo* use of HI-TOPK-032 seemed to imply a potential treatment for tumor reduction, we decided to investigate the relative effects on tumor cells compared with NSCs from the brain and their differentiated counterparts. Tumor-derived stem cells from 22 GBM patients were cultured from primary tumor tissue samples. These were cultured in *VML* (serum free) media and *Failsafe* media (1 % serum + mitogens). 18 cultures thrived in *VML* conditions while all 22 thrived in *Failsafe*. Once cultures were established they were incubated for three days in media containing HI-TOPK-032 inhibitor at 0, 1.75, 3.5 and 5 μM concentrations. Cells were plated at 50,000 cells per well. After three days cells were then harvested and total cell number, number of dead cells and net number of live cells were determined (Fig. 5 and Additional file 9: Figure S4). Six cultures of normal NSCs were grown and tested from three human biopsies as described for the tumor-derived cultures. Cells differentiated from stem cell cultures were also tested (*n* = 3) for both tumor and normal patients.

Total cell numbers were maintained in all culture types except for GICs grown adherently in *Failsafe,* which decreased in number in 5 μM of inhibitor compared to no inhibitor (*P* = 0.019, 2 tailed *T*-test). However, the number of dead cells increased in all cultures with increasing concentration of inhibitor (P < 0.05) (Additional file 9: Figure S4).

Therefore, results for net live cells were analyzed in more detail. Using two-way Anova, we assessed the interaction between concentration of inhibitor and cell type. Interaction with cell type was not statistically significant whereas concentration of inhibitor reduced net cell numbers significantly in a dose-dependent manner (*P* = 0.0031). We then tested whether normal stem cells fared better than GICs. At an inhibitor concentration of 3.5 μM both groups of tumor stem cells experienced a significant reduction in net live cells (*P*</=0.05) but reduction in normal stem cells did not reach significance (*P* = 0.17). At an inhibitor concentration of 3.5 μM differentiated cells of both normal and tumor origin exhibited no significant reduction in net live cell numbers (*P* = 0.69 and 0.67 respectively). Thus there was a slight differential tolerance to the inhibitor treatment in normal NSCs and in differentiated cells from both tumor and normal tissue sources.

## Discussion

GBM, the most prevalent and malignant primary brain tumor, is highly resistant to conventional therapies [33–35]. Tumor recurrence is the major issue in the treatment of this tumor, and has been attributed to the stem cell population in GBM [6, 36]. Through the establishment of neurospheres, GBM cells can be enriched for GICs [37], and this GIC-enriched population provides a good opportunity to identify novel therapeutic targets in malignant GBM. Several previous reports have linked increased expression of PBK to various cancers including lymphoma, leukemia, melanoma, colorectal cancer, breast cancer, lung cancer and cholangiocarcinoma [21, 24, 28, 29, 31, 38], but PBK expression has not been reported in GBM.

We recently compared *PBK* gene expression in GICs to that in NSCs (12) and subsequently found that *PBK* expression was upregulated in GICs from all patients investigated (Stangeland *et al.,* submitted). This was confirmed at the protein level and it was further shown that the protein expression of PBK in GBM tissues was high relative to normal cortical tissues (Stangeland *et al.,* submitted).

The strong and consistent upregulation of PBK in GICs prompted us to test whether PBK is involved in the maintenance of key tumorigenic properties. To do this, we used RNAi technology and pharmacological inhibition of PBK in three GIC cultures that had particularly high levels of PBK protein. Both treatments led to substantial decrements in viability and sphere formation in GIC cultures, and pharmacological inhibition also caused a substantial increase in apoptosis. Thus, PBK emerges as a particularly interesting potential therapeutic target for arresting the growth of GBM.

RNAi technology has been widely used in mammalian cells to suppress the expression of a variety of genes and thereby facilitates the definition of the functional roles of genes, especially in diseases. We have delivered three shRNAs specific for *PBK* using a lentiviral system. The different shRNA constructs were able to efficiently knock down *PBK* gene expression. We found that *PBK* knockdown significantly decreased viability and sphere forming ability in GIC cultures.

To further validate PBK’s role in GIC growth and survival, we treated different GIC cultures with a pharmacological inhibitor used previously to target PBK in colon cancer cells [28]. Different GICs treated with two different concentrations of this inhibitor show a marked, dose-dependent reduction in viability and sphere formation. Of particular note is the huge increase in apoptosis obtained using this method, which was not observed with shRNA treatment. This probably reflects the different modes of action of shRNAs and pharmacological inhibition, the latter having the more direct effect on the protein target. Analysis of the downstream targets ERK1/2 and p38 MAP kinase showed a marked reduction of phosphorylated forms of these proteins in both HI-TOPK-032 treated cells and knockdown cultures. Decrement in phospho ERK1/2 and phospho p38 levels correlated well with the reduction in cell viability in HI-TOPK-032 treated cells (Fig. 3a, e-f). These results are in agreement with studies in other cancers [17] and further emphasize the role of PBK signalling in tumor cell proliferation.

Our findings are similar to those of Kim and co-workers [28], who showed that the administration of HI-TOPK-032 at a dose of 1 and 10 mg/kg bodyweight as an experimental therapy for pre-established subcutaneous colon cancer xenografts led to a massive reduction in tumor volume. We find that HI-TOPK-032 at doses of 5 and 10 mg/kg markedly attenuates the growth of pre-established subcutaneous GBM xenografts. Notably, our immunohistological investigation of the HI-TOPK-032-treated tumors revealed a decrease in proliferating cells accompanied by a slight increase in the number of apoptotic cells.

Having established a significant role for PBK in three gliomas we set out to test whether inhibition of PBK would affect a large number of tumors and to gauge how well normal cells fare under a treatment scenario. Twenty-two out of 22 primary tumor cell lines were severely reduced in net live cell numbers after three days of treatment, and cell death was pronounced in all samples. Normal NSCs seemed to withstand the treatment better at 3.5 μM than GICs. Both normal- and tumor-derived differentiated cells fared better than GICs. In summary, our results support an effect of the inhibitor when given directly into the tumor. The possibility of systemic administration should be investigated when new inhibitors have been developed.

## Conclusions

Our observations *in vitro* and *in vivo* confirm the functional importance of PBK in the growth and survival of GBM cells. PBK may therefore serve as a potential therapeutic target in GBM tumors.

## Methods

### Cell culture

Tumor biopsies were obtained from consenting patients as approved by the Norwegian National Committee for Medical Research Ethics. Briefly, primary GBM tissue samples were washed, and enzymatically dissociated to single cells by incubation in Trypsin-EDTA (Invitrogen). Trypsin treatment was blocked by adding 2 mg/ml human albumin (Octapharma Pharmazeutika Produktiones, Austria) and the cells were then washed in Leibowitz-15 medium (L-15, Invitrogen). The cells were resuspended as single cells and cultured in a chemically defined serum-free neurosphere medium consisting of 10 ng/ml basic fibroblast growth factor (bFGF) and 20 ng/ml epidermal growth factor (EGF) (both R&D Inc., Minneapolis, MN), B27-supplement (1:50, Invitrogen), 100 U/ml Penicillin/streptomycin (Lonza, Switzerland), 1 ng/ml Heparin (Leo Pharma, Denmark) and 8 mM Hepes (Lonza) in Dulbecco’s modified essential medium with nutrient mix F-12 and Glutamax (DMEM/F12, Invitrogen) (*‘VML’* medium). Cells were cultured at a density of 10^5^ cells/ml in 75 cm^2^ non-treated cell culturing flasks. GIC cultures were characterized for the following stemness markers- CD133, CD44, SSEA-1/CD15, CXCR4, CD9, CD166, A2B5 (Additional file 10: Figure S5). GIC cultures were routinely characterized for their tumor forming capability by transplanting them into the brain of SCID mice (Additional file 11: Figure S6).

Adherent NSCs were grown in modified neurosphere medium containing 1 % FBS, 10 ng/ml bFGF and 20 ng/ml TGF α (‘*Failsafe medium’*) [39]. NSCs were isolated from SVZ and hippocampus.

Cells of the ReNcell VM Human Neural Progenitor Cell Line (Millipore, SCC008) were grown as spheres in serum free Neurobasal A medium (Life technologies, 10888–022) containing B27 (Gibco-BRL, Rockville, MD, USA), 10 ng/ml basic fibroblast growth factor, and 20 ng/ml epidermal growth factor (both from R&D Systems, Minneapolis, MN, USA).

### In vitro differentiation

Dissociated cells (tumor-derived and normal NSCs) were plated at 50 000 cells/well in 6 well plates (Nunc, VWR Norge) coated with 15 μg/ml poly-L-ornithine (Sigma, St. Louis, MO). DMEM/F12 medium was augmented with fetal calf serum (3.75 %; PAA Laboratories, Pasching, Austria) and 25 μl/ml B-27 with vitamin A (Invitrogen), 1 μg/ml laminin and 10 μl/ml of Pen/Strep. All cultures were kept in an incubator at 5–6 % CO_2_, pH 7.2-7.4 [40].

### RNA isolation and real-time quantitative reverse-transcription PCR (qPCR)

Total RNA from cells (2×10^6^) was isolated using the RNeasy Mini Kit (Qiagen). The concentration of each RNA sample was determined by using the Nanodrop spectrophotometer. cDNA was synthesized from 1 μg of RNA using a QuantiTect Reverse Transcription kit (Qiagen). qPCR was performed on a ABI PRISM 7900HT (Applied Biosystems) with SYBR® *Premix Ex Taq*™ (Perfect Real Time) (Takara) according to the manufacturer's instructions. Experiments were performed on at least three biological replicates (different passages). Crossing point (CP) values were generated using second-derivative calculation software (SDS2.2). Expression levels were calculated using REST software [41]. The primers for qPCR were designed according to Vector NTI-based Web service for primer design: Probe Wiz Server, Center for Biological Sequence Analysis [42]. The oligonucleotide sequences used for PBK expression analyses were:

Oli_7_DIR_PBK_set1_1515_TGGATCTACTGACATTAGCACTTTGTA, Oli_8_REV_PBK_set1_1844_CCAAAGTGTCCTTTATTCTTTATCATC, Oli_9_DIR_PBK_set2_904_TTACTTTGTGGGAAATGATGACTTTAT, Oli_10_REV_PBK_set2_1107_CATTAGTGCATACAGAGAAGAGTTCAA.

### Western blot

The cells (2-6 × 10^6^) were trypsinized, washed with PBS and homogenized by triturating in Cell Extraction (CE) Buffer (Mammalian cell extraction kit K269-500, Biovision). The homogenates were then vortexed for a few seconds, incubated on ice for 10 min and finally centrifuged at max speed (microfuge) for 1 min through a QIAshredder (Qiagen). The supernatants were collected, and the amount of total protein was determined using the BCA protein assay kit (Thermo Scientific). 20–40 μg of whole protein extracts were mixed with the loading buffer (NuPAGE) and loaded onto a 4-12 % gradient Nu-PAGE gel (Invitrogen). Protein gels were blotted onto a 0.45 μm PVDF membrane. The membrane was blocked with 5 % skimmed milk in TBS/0.1 % Tween 20 (TBST) and probed with primary antibodies in TBST and 5 % skimmed milk. Secondary antibodies were HRP-conjugated anti-rabbit IgG, anti-mouse IgG and anti-goat IgG (1/10 000 in 5 % skimmed milk in TBST). The blots were developed using Lumiglo Reserve CL Substrate kit, detected by Kodak Molecular Imaging System (Kodak MI, version 5.0) or the Epi Chemi II Darkroom (UVP-Laboratory Products) and Labworks software (UVP). Western blot was performed using rabbit anti-PBK [#4942 (Cell Signaling), 1:1000], Phospho ERK 1 and 2 [#9101 (Cell Signaling), 1:1000], Total ERK 1 and 2 [#9102 (Cell Signaling), 1:1000], Phospho-p38 MAP Kinase (Thr180/Tyr182) [#9216 (Cell Signaling) (mouse) 1:1000] and ACTIN [#4967 (Cell Signaling), 1:1000] antibodies. For secondary antibody we used ECL Anti-rabbit IgG-HRP [NA934 (Amersham), 1:10000]. To calculate relative protein expression (RPE) the intensities of the protein bands from Western blots were quantified using Adobe Photoshop and normalized to the intensities of the corresponding β-actin (ACTB) bands. The RPE values were used for calculation of the Pearson product–moment correlation coefficient *r*.

### Flow cytometry

Flow cytometry analysis of surface markers was performed as previously described [43]. Intracellular staining was performed by using Fixation/Permeabilization Solution Kit (BD Pharmingen), where cells were incubated with primary antibody over night, followed by washing and then incubated with secondary antibody for 2 h. Cells stained with secondary antibodies alone were used as control for gating. The following antibodies were used: CD166-PE, CD9- FITC, CXCR4-PE, m/hCD44-APC (eBioscience, San Deigo, CA), CD133/2-PE and anti-m/hAPC-A2B5 (Miltenyi, Billerica, MA).

### Immunolabeling and confocal microscopy

Tumor spheres were spun down, fixed in 4 % paraformaldehyde, cryo-protected in 20 % sucrose in PBS, cryo-embedded in OCT (Tissue-TEK, Sakura Finetek, CA) and immunostained as described in [44, 45]. GBM tissue samples, cortex from the normal brain, and HI-TOPK-032 treated tissue from the subcutaneous tumors were fixed, cryo-protected and cryo-embedded in a similar way. Sections of 12 μm were made with a cryostat, mounted on Super Frost (Thermo scientific) microscope slides and were immunostained [44].

Immunostaining was done using the following primary antibodies: anti-PBK [#612170 (BD Transduction Laboratories), Mouse, 1:100], anti-Ki67 [#SC15402 (Santa Cruz), Rabbit, 1:200] and anti-Cleaved Caspase-3 [#9661 (Cell Signaling), Rabbit, 1:400]. Secondary antibodies used were goat anti-Mouse and goat anti-Rabbit Cy3 conjugates (Amersham Pharmacia Biosciences). Cells and sections were fluorescence counterstained with Hoechst 33342 and mounted in 50:50 PBS: glycerol. Negative and positive controls were included in each experiment. Confocal images were acquired with a Zeiss Meta 510 confocal laser scanning microscope.

### Lentiviral mediated shRNA knockdown

Three different GIC cultures were used to establish *PBK* knockdown cell lines each with three different shRNA constructs (RMM4431-98972202, RHS4430-101071930, RHS4430-101072940, Thermo Scientific, Open Biosystems). Nine μg of plasmid DNA were transfected into the 293FT cell line using Arrest-In transfection reagent (according to the manufacturer’s protocol). Briefly, viral supernatants collected after 48 and 72 h were centrifuged at 3000 rpm for 20 min at 4 °C and filtered through a sterile 0.45 μm low protein binding filter (Sarstedt). The virus was then concentrated in sterile SW28 ultracentrifuge tubes by ultracentrifugation (Beckman Optima™ LE-80 K ultracentrifuge) equipped with a SW-28 rotor at 23,000 rpm for 1.5 h at 4 °C. The pellet was resuspended in 200 μl of DMEM and aliquots of the concentrated virus were stored at −80 °C. Tumor cells (10^5^ cells/well) were then transduced in 24 well plates by adding 10 μl concentrated virus/well and the cells were incubated for 48 h at 37 °C in 5 % CO_2_. Three to five days after transduction, GFP positive cells were purified by FACS sorting using a FACS Diva cell sorter equipped with an argon ion laser, ‘TurboSort Plus’ option, and Diva software (Becton Dickinson). The cells were then selected in 2 μg/ml Puromycin (Sigma, USA) for 3–4 weeks and used for functional assays.

### PBK/TOPK inhibitor

The PBK/TOPK inhibitor HI-TOPK-032 was purchased from Inter BioScreen, Russia. For the functional assays done on HI-TOPK-032 treated cells, cells were first incubated with the inhibitor at a concentration of 5 μM and 10 μM for 3 days. This HI-TOPK-032 is expected to bind to the active site of PBK [28]. Stock concentration (1 mM) of HI-TOPK-032 was dissolved in 1 ml of DMSO.

### Cell viability assay

A colorimetric test based on tetrazolium salt-XTT conversion (Roche Diagnostics, Indianapolis, IN) was used to measure the viability in control, PBK knockdown cell lines and HI-TOPK-032 treated cells, according to the manufacturer’s instructions. Commonly referred to as a cell viability assay, this actually reflects all aspects of cell activity including cell growth, cell proliferation, and any changes in metabolic state in the absence of growth and proliferation. Briefly, cells were plated at a density of 1 × 10^4^ cells per well of a 96-well plate. We measured five wells for each cell sample. The colorimetric changes (absorbance) were measured after 24 h at 490 nm using a plate reader (Victor™ X5, Perkin Elmer 2030 Multi-label reader) after the addition of XTT reagent (50 μl/well).

### Sphere-forming assay

Spheres (control, PBK knockdown cell lines and HI-TOPK-032 treated cells) were dissociated into single cell suspensions and 500 cells per well were plated in ultra-low attachment 96-well plates (Sarstedt, USA). The number and diameter of spheres in 10 wells were measured for each cell sample. The formation of spheres per well was evaluated 10 days after commencement of culturing them. Only the spheres above 50 μm were taken into consideration. The number and the size of spheres (average area) were measured by using GelCount™ (Oxford Optronix, UK).

### Apoptosis

The Homogeneous Caspases Assay, which is a fluorimetric test used for the quantitation of caspase activity *in vitro* (Roche Diagnostics GmbH, Germany) was used to determine the rate of apoptosis in control and PBK knockdown cell lines, and HI-TOPK-032 treated cells, according to the manufacturer’s instructions. Briefly, control and PBK knockdown cell lines were plated at a density of 5 × 10^3^ cells per well of a 96-well plate. Three wells were measured for each cell sample. The fluorimetric changes were measured after 24 h at 485/535 nm using a plate reader (Victor™ X5, Perkin Elmer 2030, Multilabel reader) after the addition of substrate (100 μl/well).

### Microarray hybridization and analysis of microarray data

RNA was amplified and hybridized to Illumina Human HT-12 v4 microarrays. Analysis and statistics were done using J-Express (Molmine) and Web Gestalt [46]. Microarrays were quantile normalized and differential gene analysis carried out using Rank product analysis [47]. Pathways and gene ontology were obtained from KEGG [48], Wiki pathways [49] and Gene Ontology [50]. Microarray data from our lab are MIAME compliant and the following link has been created to allow review of the data in Gene Expression Omnibus [51] (GEO accession number is GSE53800): https://www.ncbi.nlm.nih.gov/geo/query/acc.cgi?acc=GSE53800.

### Xenograft mouse model for testing of HI-TOPK-032

All animal procedures were approved by the National Animal Research Authority (NARA #4799). C.B.-17 severely combined immunodeficient (SCID) male mice (8–9 weeks old) were obtained from Taconic (Ejby, Denmark). They were maintained in a minimal disease unit under “specific pathogen-free” conditions according to the guidelines by the NARA and acclimatized for >1 week. Prior to the inoculation of cells, mice were anesthetized with a subcutaneous injection of Hypnorm (10 mg/mL fluanisone and 0.315 mg/mL fentanyl citrate; Veta Pharma) and Dormicum (5 mg/mL midazolam; Roche). Mice were divided into three groups: (i) control group (*n* = 4), (ii) HI-TOPK-032 5 mg/kg of bodyweight (*n* = 4) and (iii) HI-TOPK-032 10 mg/kg of bodyweight (*n* = 4).

GICs from T08 expressing GFP were suspended in neurosphere medium and 1:4 Matrigel (Basement membrane matrix, BD Biosciences) and 1.5 × 10^6^ cells/100 μl were inoculated subcutaneously into the right flank of each mouse. When tumors started to become visible the mice were given treatment three times weekly for 28 days with intra-tumoral injections according to their assigned groups. Stock concentration (100 mM) of HI-TOPK-032 in DMSO was diluted in physiological NaCl (9 mg/ml; Braun) and the control group received an equivalent amount of NaCl solution. All animals were monitored for adverse effects especially due to inhibitor administration and examined regularly for general appearance, signs of distress (local and systemic toxicity), and with weight measurements.

All mice were sacrificed when tumors became > 1.5 cm in diameter. Tumors were extracted and measurements obtained for 3 planes. Tumor volumes were calculated using the formula for a triaxial ellipsoid (4/3xr1xr2xr3xπ). Xenograft tumors were fixed overnight in 10 % buffered formalin, cryoprotected in 20 % sucrose for 24 h and then cryo-embedded in OCT (Tissue-TEK, Sakura Finetek, CA) and stored at −20 °C. Tumors were cut into 12 μm sections for immunolabeling as described previously.

### Effects on wide range of GBM patient samples: In vitro simulation of treatment with inhibitor

Cells were seeded at 50,000 cells per well and HI-TOPK-032 inhibitor included in the culture media at 0, 1.75, 3.5 and 5 μM in DMSO vehicle (15 μL). Total and dead cells were counted after three days using a *Nucleocounter* (Chemotetec). Total and net live cells were expressed as fold of the cell numbers for vehicle only. Dead cells were expressed as % of total cell numbers.

### Statistical tests

Statistical evaluation of the results obtained in the functional assays (proliferation, sphere forming ability, apoptosis) was done using the non-parametric Mann–Whitney *U*-test for two unpaired groups. For the *in vitro* inhibition tests Two Way Anova was applied for interaction of Inhibitor Concentration and Cell Type. For comparison of individual means for concentration effects, a two tailed Student’s *T*-test was applied.

## Additional files

Additional file 1: Table S1. This table shows P values and fold change expression of *PBK* by qPCR in different GICs patient samples relative to NSCs control cells. Additional file 2: Figure S1. Confirmation of *PBK* knockdown by qPCR. *PBK* mRNA was quantified in three GIC lines treated with shRNAs 1, 2 and 3 compared to the Non-silencing controls. Relative expression of *PBK* confirmed efficient knockdowns in the following cultures: T65 treated with shRNAs 2 and 3, T08 treated with all three shRNAs and T59 treated with shRNAs 1 and 2. Additional statistical parameters are shown in Additional file 3: Table S2. Additional file 3: Table S2. The statistical values to show the relative expression of *PBK* in shRNA lines (as shown in Additional file 2: Figure S1) are presented in this table. Additional file 4: Figure S2. Induction of cell death by *PBK* knockdown. Apoptosis measured as increase in Caspase-3 activity is evident in all KD lines from T08 GIC cultures but in none of the KD lines from T65 and T59 GIC cultures. Error bars = SD, *n* = 3. The statistical analysis was performed using unpaired *T*-test with Welch’s correction. The asterisks indicate the level of significance (* ≈ p ≈ 0.01-0.05). The values obtained for knockdown cultures were compared to the values obtained for NS controls. Additional file 5: Figure S3. Pharmacological treatment of KD lines with HI-TOPK-032 reduces viability and sphere forming capacity. One KD line (shRNA 3) from each of T65, T08 and T59 cell lines together with the respective Non-silencing control cells exhibited reduced viability (A) and sphere formation (B), sphere size (C) and increased apoptosis (D) when treated with HI-TOPK-032 (5 μm). All values shown are normalized to the measurements made in the respective Non-silencing control cultures. Error bars = SD, *n* = 3. The statistical analysis was performed using unpaired *T*-test with Welch’s correction. The asterisks indicate the level of significance (* ≈ p ≈ 0.01-0.05). The values obtained for hybrid treatment (shRNA + inhibitor) were compared to values obtained for shRNA. Additional file 6: Figure S7.
*In vitro* dilution assay performed on T08 (untreated and treated with HI-TOPK-032 - 5 μm) shows a similar decrease in the percentage of clonally derived spheres as seen before. We also performed this assay on the normal NFCs under similar conditions. NFCs survived the treatment slightly better than the T08 cells. Error bars = SD, *n* = 2 for T08 and *n* = 3 for NFCs. Additional file 7: Table S3. Gene expression analysis of *PBK* knockdown shRNA cell lines (T08 shRNA2, shRNA3 and T65 shRNA2 shRNA3) and PBK inhibitor treated (T65, T08, T59 – all treated with 5 μM). Additional file 8: Figure S8. Western blot showing phosphorylation status of the p38 MAP Kinase (Thr180/Tyr182) in the GIC cultures featuring *PBK* knockdown, original GIC cultures and one NSC culture. The relative expression values were calculated using NS as a control (except for T65 where the original culture was used as a reference). Additional file 9: Figure S4. Dead cells expressed as percentage of total cell numbers for cultures for HI-TOPK-032 at various concentrations. There was an increase of dose dependent cell death with concentration in all cultures (p < 0.05; A: *n* = 22; B: *n* = 18; C: *n* = 6; D: *n* = 3; E: *n* = 3. Error bars = SEM). Additional file 10: Figure S5. Immunophenotypic characterization of GICs shows expression of surface stem cells markers and proliferation marker. Most of the cells from the three different patients were highly positive for CD44, CD166, and CD9 in the GICs. On the other hand, CXCR4, A2B5, CD133, and SSEA1/CD15 vary among patients. The proliferation marker Ki67 was expressed in 55 % of the cells in T08 and 53 % in T65. (−*) data not available. Additional file 11: Figure S6. GIC cultures T08 (A) and T65 (B) were transduced with lentiviral vectors expressing GFP and transplanted intracranially to SCID-mice. Representative images showing invasive tumors formed upon xenotransplantation. Scale bar is 1 mm.

## Abbreviations

GBM: Glioblastoma multiforme
GIC: Glioma initiating cell
PBK: PDZ-binding kinase
TOPK: T-LAK cell-originated protein kinase
MAPK: Mitogen-activated protein kinase
NSCs: Neural stem cells
shRNA: short hairpin RNA
