# Supplementary material for: Targeting PBK/TOPK decreases growth and survival of glioma initiating cells in vitro and attenuates tumor growth in vivo
Source: Mol Cancer. 2015 Jun 17;14:121. doi: 10.1186/s12943-015-0398-x (PMC4470057; doi:10.1186/s12943-015-0398-x)
Supplement: Additional file 1: Table S1. — This table shows P values and fold change expression of PBK by qPCR in different GICs patient samples relative to NSCs control cells. [file 12943_2015_398_MOESM1_ESM.pdf]

GBM culture	Fold expression	Std. Error	95% C.I.	P(H1)	Result
fNPCs	3,86	1,459-8,245	0,796-15,203	0,057	
T65	26,765	17,266-55,678	8,903-73,714	0	UP
CAST 3	27,131	22,189-33,507	19,765-36,902	0,024	UP
T08	43,583	7,866-181,984	5,143-242,061	0	UP
CAST4	25,346	22,623-28,683	20,152-31,589	0,028	UP
T96	48,294	8,348-179,194	7,392-210,374	0	UP
T111	18,842	3,229-72,695	2,783-83,822	0	UP
T59	23,122	3,974-74,070	2,685-148,621	0,001	UP

Supplementary Table -1
