# Supplementary material for: Targeting PBK/TOPK decreases growth and survival of glioma initiating cells in vitro and attenuates tumor growth in vivo
Source: Mol Cancer. 2015 Jun 17;14:121. doi: 10.1186/s12943-015-0398-x (PMC4470057; doi:10.1186/s12943-015-0398-x)
Supplement: Additional file 2: Figure S1. — Confirmation of PBK knockdown by qPCR. PBK mRNA was quantified in three GIC lines treated with shRNAs 1, 2 and 3 compared to the Non-silencing controls. Relative expression of PBK confirmed efficient knockdowns in the following cultures: T65 treated with shRNAs 2 and 3, T08 treated with all three shRNAs and T59 treated with shRNAs 1 and 2. Additional statistical parameters are shown in Additional file 3: Table S2. [file 12943_2015_398_MOESM2_ESM.pdf]

Relative Expression of PBK in T65 shRNA lines

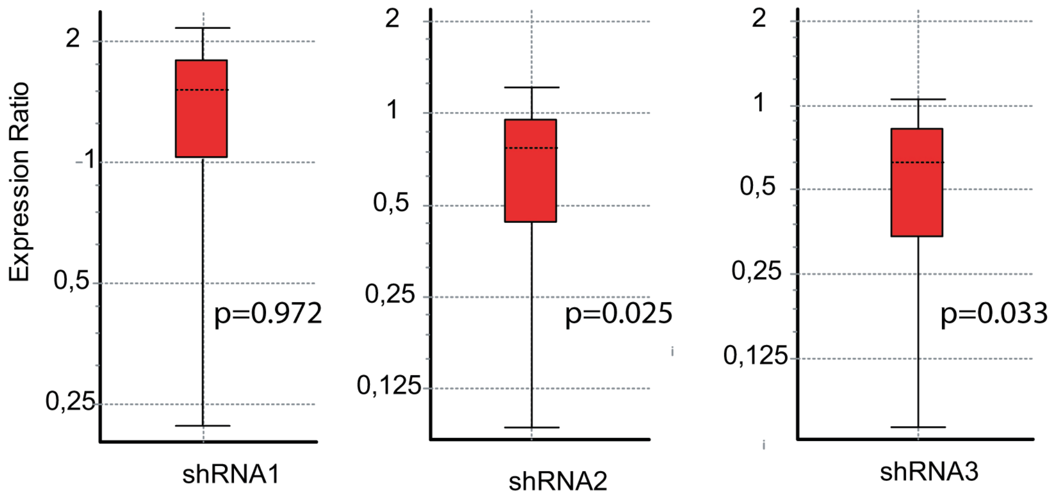

Relative Expression of PBK in T08 shRNA lines

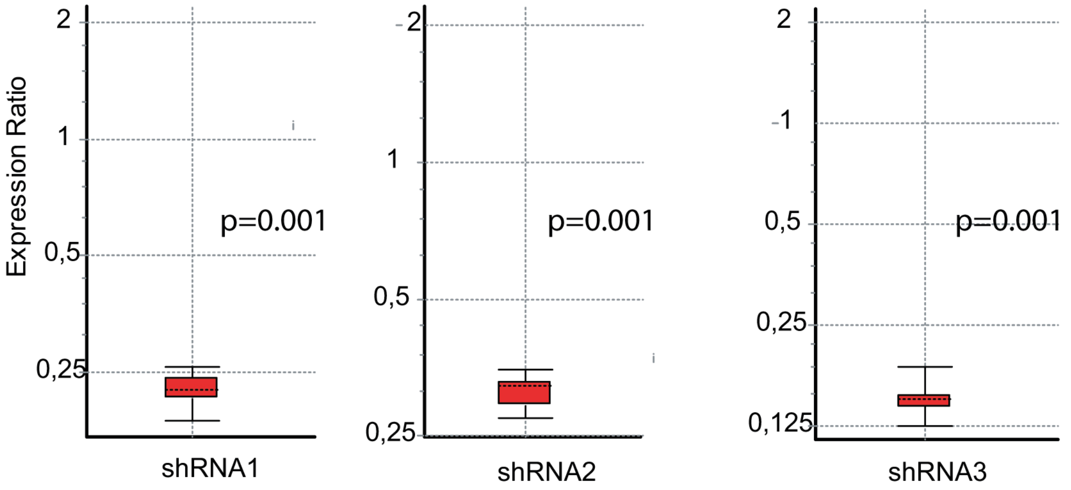

Relative Expression of PBK in T59 shRNA lines

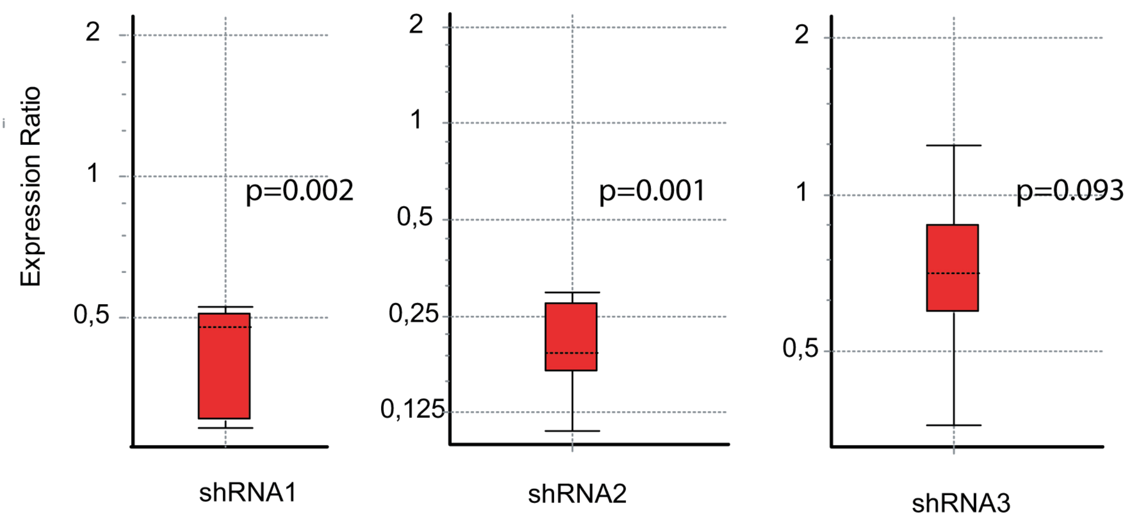

Supplementary figure- 1
