# Supplementary material for: Targeting PBK/TOPK decreases growth and survival of glioma initiating cells in vitro and attenuates tumor growth in vivo
Source: Mol Cancer. 2015 Jun 17;14:121. doi: 10.1186/s12943-015-0398-x (PMC4470057; doi:10.1186/s12943-015-0398-x)
Supplement: Additional file 3: Table S2. — The statistical values to show the relative expression of PBK in shRNA lines (as shown in Additional file 2: Figure S1) are presented in this table. [file 12943_2015_398_MOESM3_ESM.pdf]

	Expression	Std. Error	95% C.I.	P(H1)	Result
**T65**					
shRNA1	0,994	0,255-1,807	0,222-2,058	0,972	
shRNA2	0,527	0,138-1,034	0,093-1,223	0,025	**DOWN**
shRNA3	0,434	0,124-0,926	0,071-1,053	0,033	**DOWN**
**T08**					
shRNA1	0,223	0,203-0,243	0,187-0,257	0,001	**DOWN**
shRNA2	0,313	0,284-0,340	0,274-0,350	0,001	**DOWN**
shRNA3	0,313	0,284-0,340	0,274-0,350	0,001	**DOWN**
**T59**					
shRNA1	0,419	0,292- 0,513	0,291- 0,528	0,002	**DOWN**
shRNA2	0,207	0,167-0,294	0,109- 0,296	0,001	**DOWN**
shRNA3	0,717	0,500- 1,158	0,361- 1,240	0,093	

Supplementary Table -2
