# Supplementary figures and images for: Targeting PBK/TOPK decreases growth and survival of glioma initiating cells in vitro and attenuates tumor growth in vivo
Source: Mol Cancer. 2015 Jun 17;14:121. doi: 10.1186/s12943-015-0398-x (PMC4470057; doi:10.1186/s12943-015-0398-x)

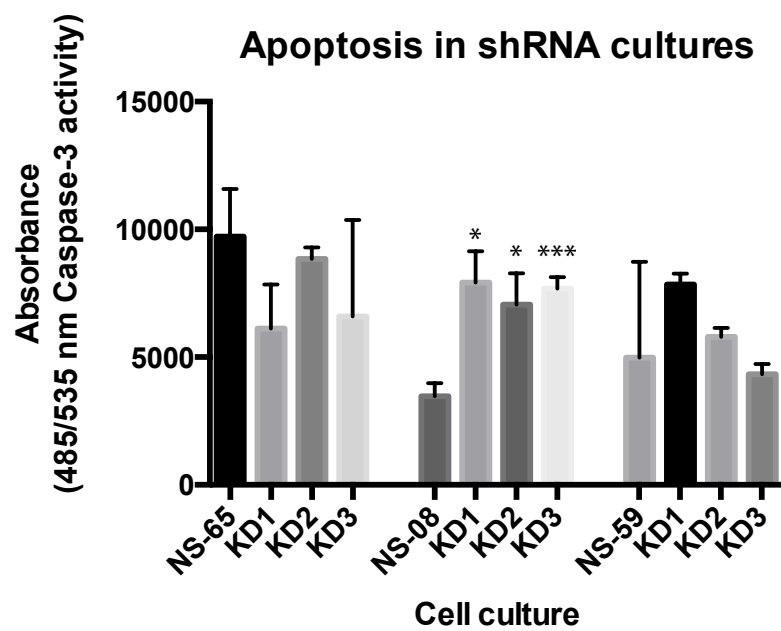

Supplementary Figure 2

Supplement: Additional file 4: Figure S2. — Induction of cell death by PBK knockdown. Apoptosis measured as increase in Caspase-3 activity is evident in all KD lines from T08 GIC cultures but in none of the KD lines from T65 and T59 GIC cultures. Error bars = SD, n = 3. The statistical analysis was performed using unpaired T-test with Welch’s correction. The asterisks indicate the level of significance (* ≈ p ≈ 0.01-0.05). The values obtained for knockdown cultures were compared to the values obtained for NS controls. [file 12943_2015_398_MOESM4_ESM.pdf]

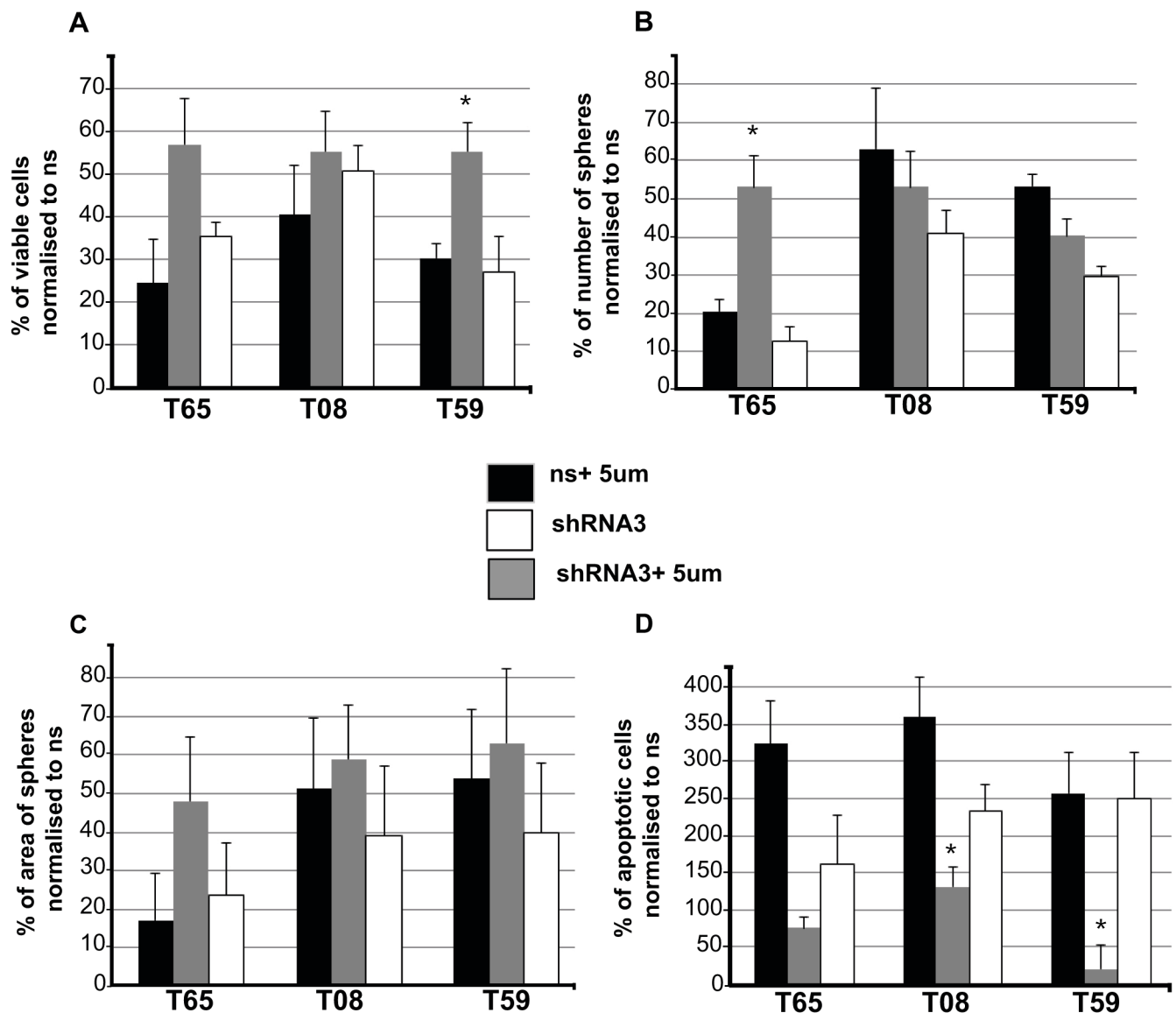

Supplementary figure- 3

Supplement: Additional file 5: Figure S3. — Pharmacological treatment of KD lines with HI-TOPK-032 reduces viability and sphere forming capacity. One KD line (shRNA 3) from each of T65, T08 and T59 cell lines together with the respective Non-silencing control cells exhibited reduced viability (A) and sphere formation (B), sphere size (C) and increased apoptosis (D) when treated with HI-TOPK-032 (5 μm). All values shown are normalized to the measurements made in the respective Non-silencing control cultures. Error bars = SD, n = 3. The statistical analysis was performed using unpaired T-test with Welch’s correction. The asterisks indicate the level of significance (* ≈ p ≈ 0.01-0.05). The values obtained for hybrid treatment (shRNA + inhibitor) were compared to values obtained for shRNA. [file 12943_2015_398_MOESM5_ESM.pdf]

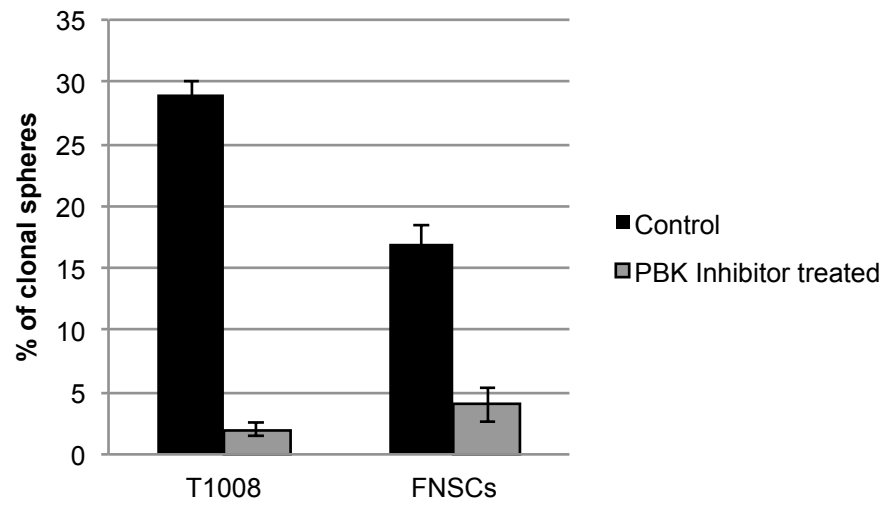

**Supplementary Figure 7**

Supplement: Additional file 6: Figure S7. — In vitro dilution assay performed on T08 (untreated and treated with HI-TOPK-032 - 5 μm) shows a similar decrease in the percentage of clonally derived spheres as seen before. We also performed this assay on the normal NFCs under similar conditions. NFCs survived the treatment slightly better than the T08 cells. Error bars = SD, n = 2 for T08 and n = 3 for NFCs. [file 12943_2015_398_MOESM6_ESM.pdf]

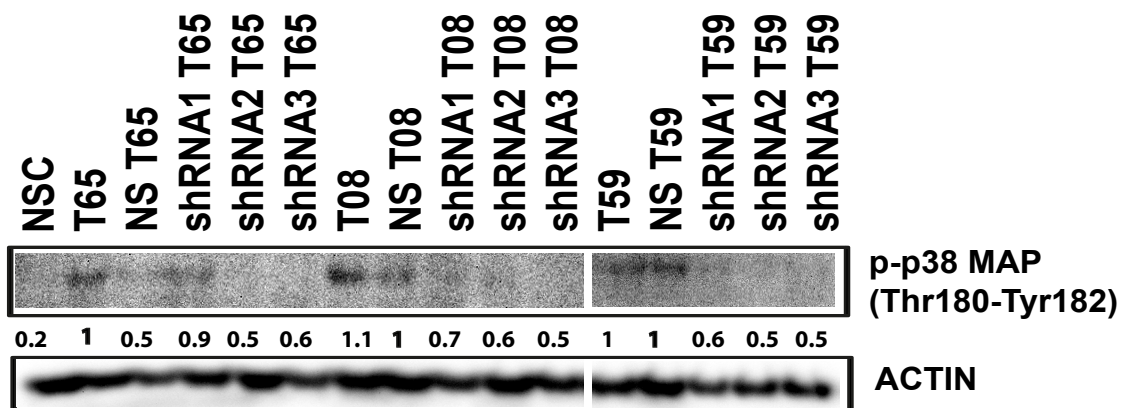

Supplementary Figure 8

Supplement: Additional file 8: Figure S8. — Western blot showing phosphorylation status of the p38 MAP Kinase (Thr180/Tyr182) in the GIC cultures featuring PBK knockdown, original GIC cultures and one NSC culture. The relative expression values were calculated using NS as a control (except for T65 where the original culture was used as a reference). [file 12943_2015_398_MOESM8_ESM.pdf]

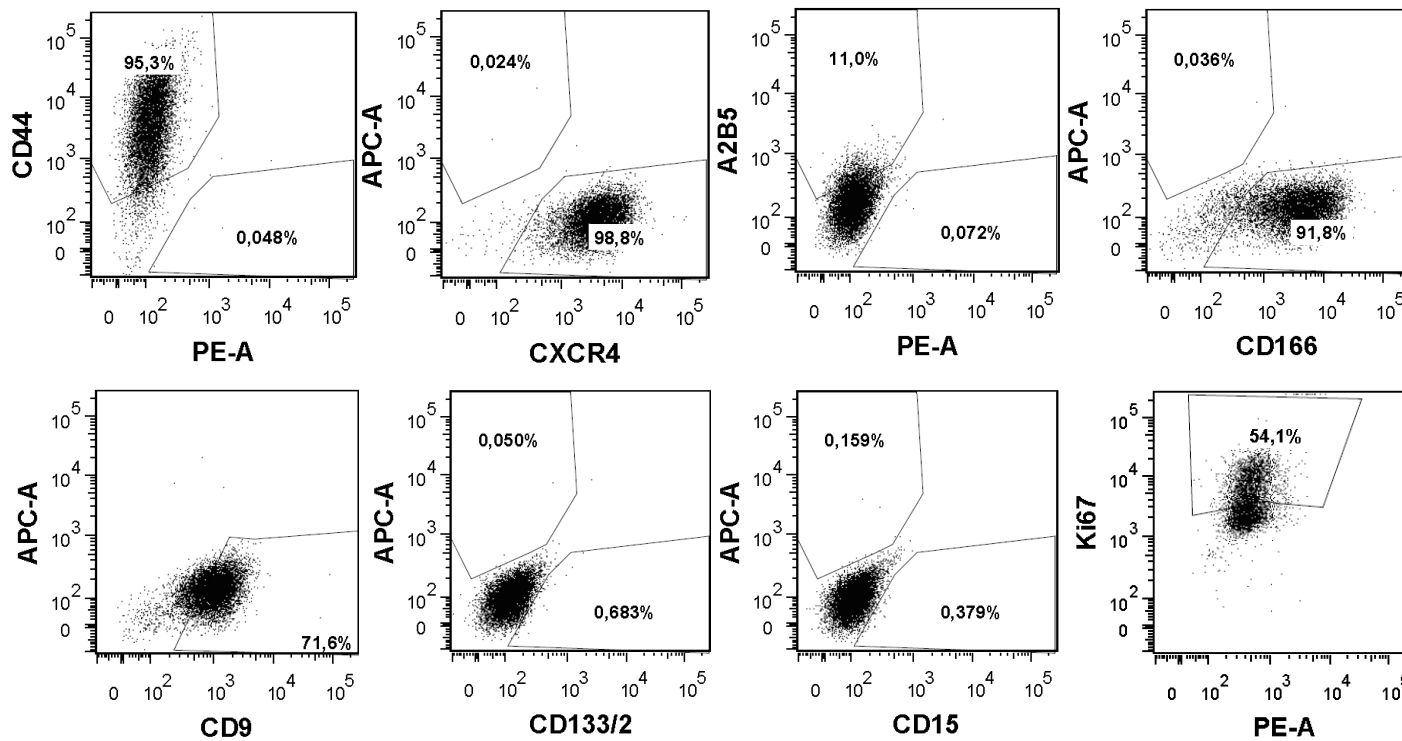

| Tumor | CD44 | CXCR4 | A2B5 | CD166 | CD9 | CD133 | CD15 | Ki67 |
|-------|------|-------|------|-------|-----|-------|------|------|
| T08   | 81%  | 31%   | 34%  | 58%   | 73% | 54%   | 52%  | 55%  |
| T65   | 95%  | 97%   | 11%  | 90%   | 69% | 0%    | 0%   | 53%  |
| T59   | 70%  | 74%   | 66%  | 63%   | 11% | 0%    | 6%   | -*   |

**Supplementary Figure 5**

Supplement: Additional file 10: Figure S5. — Immunophenotypic characterization of GICs shows expression of surface stem cells markers and proliferation marker. Most of the cells from the three different patients were highly positive for CD44, CD166, and CD9 in the GICs. On the other hand, CXCR4, A2B5, CD133, and SSEA1/CD15 vary among patients. The proliferation marker Ki67 was expressed in 55 % of the cells in T08 and 53 % in T65. (−*) data not available. [file 12943_2015_398_MOESM10_ESM.pdf]

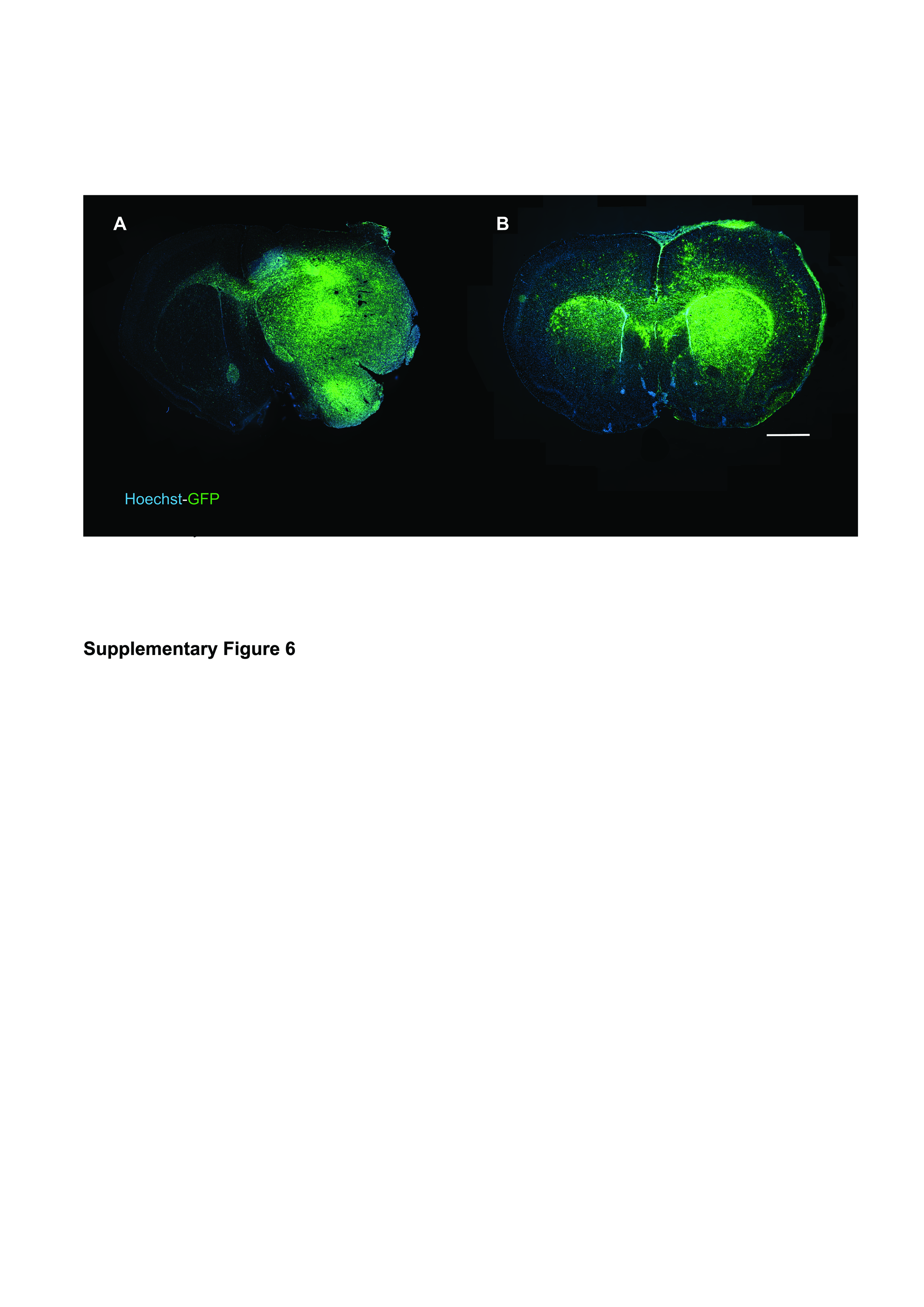

Supplement: Additional file 11: Figure S6. — GIC cultures T08 (A) and T65 (B) were transduced with lentiviral vectors expressing GFP and transplanted intracranially to SCID-mice. Representative images showing invasive tumors formed upon xenotransplantation. Scale bar is 1 mm. [file 12943_2015_398_MOESM11_ESM.tiff]
