# Supplementary material for: Targeting PBK/TOPK decreases growth and survival of glioma initiating cells in vitro and attenuates tumor growth in vivo
Source: Mol Cancer. 2015 Jun 17;14:121. doi: 10.1186/s12943-015-0398-x (PMC4470057; doi:10.1186/s12943-015-0398-x)
Supplement: Additional file 9: Figure S4. — Dead cells expressed as percentage of total cell numbers for cultures for HI-TOPK-032 at various concentrations. There was an increase of dose dependent cell death with concentration in all cultures (p < 0.05; A: n = 22; B: n = 18; C: n = 6; D: n = 3; E: n = 3. Error bars = SEM). [file 12943_2015_398_MOESM9_ESM.pdf]

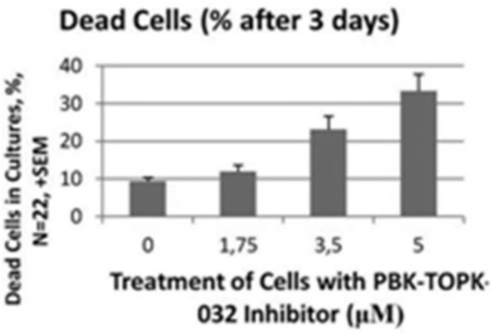

A. Tumor cells adherent

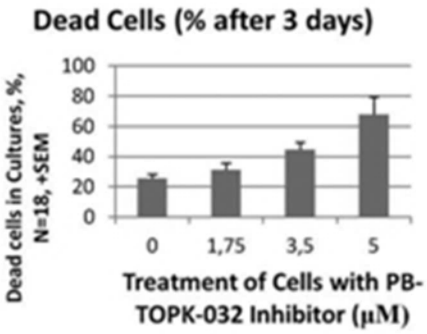

B. Tumor cells in spheres

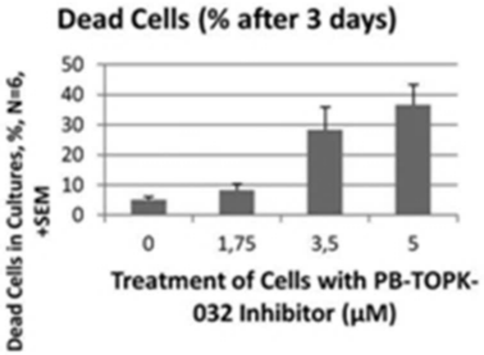

C. Normal cells adherent

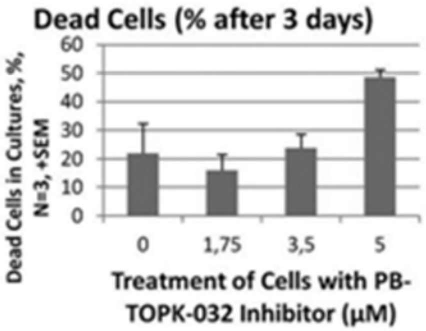

D. Normal cells differentiated

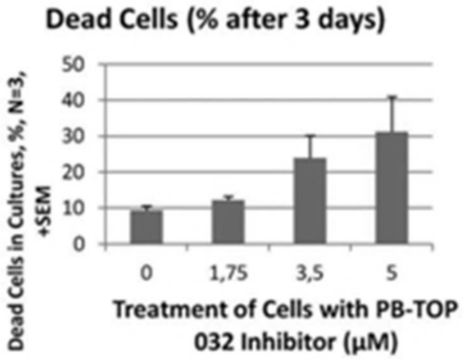

E. Tumor cells differentiated

Supplementary Figure 4
